# Redox effects and cytotoxic profiles of MJ25 and auranofin towards malignant melanoma cells

**DOI:** 10.18632/oncotarget.4108

**Published:** 2015-05-12

**Authors:** Marijke C.C. Sachweh, William C. Stafford, Catherine J. Drummond, Anna R. McCarthy, Maureen Higgins, Johanna Campbell, Bertha Brodin, Elias S.J. Arnér, Sonia Laín

**Affiliations:** ^1^ Department of Microbiology, Tumor and Cell Biology, Karolinska Institutet, Stockholm, Sweden; ^2^ Division of Biochemistry, Department of Medical Biochemistry and Biophysics, Karolinska Institutet, Stockholm, Sweden; ^3^ Centre for Oncology and Molecular Medicine, University of Dundee, Ninewells Hospital and Medical School, Dundee, Tayside, United Kingdom; ^4^ Department of Oncology and Pathology, Karolinska Institutet, Stockholm, Sweden

**Keywords:** malignant melanoma, auranofin, vemurafenib, thioredoxin reductase 1, p53

## Abstract

Malignant melanoma is the most dangerous type of skin cancer. Although recent progress in treatment has been achieved, lack of response, drug resistance and relapse remain major problems. The tumor suppressor p53 is rarely mutated in melanoma, yet it is inactive in the majority of cases due to dysregulation of upstream pathways. Thus, we screened for compounds that can activate p53 in melanoma cells. Here we describe effects of the small molecule MJ25 (2-{[2-(1,3-benzothiazol-2-ylsulfonyl)ethyl]thio}-1,3-benzoxazole), which increased the level of p53-dependent transactivation both as a single agent and in combination with nutlin-3. Furthermore, MJ25 showed potent cytotoxicity towards melanoma cell lines, whilst having weaker effects against human normal cells. MJ25 was also identified in an independent screen as an inhibitor of thioredoxin reductase 1 (TrxR1), an important selenoenzyme in the control of oxidative stress and redox regulation. The well-characterized TrxR inhibitor auranofin, which is FDA-approved and currently in clinical trials against leukemia and a number of solid cancers, displayed effects comparable with MJ25 on cells and led to eradication of cultured melanoma cells at low micromolar concentrations. In conclusion, auranofin, MJ25 or other inhibitors of TrxR1 should be evaluated as candidate compounds or leads for targeted therapy of malignant melanoma.

## INTRODUCTION

Cutaneous malignant melanoma is the most dangerous type of skin cancer and incidence rates have been rising continuously over the past decades [1, 2]. Vemurafenib (Zelboraf, PLX4032) [3] is approved for the treatment of unresectable or metastatic melanoma in which the serine/threonine kinase BRAF has been mutated at Val600, as is the case in approximately 50% of patients [4]. However, drug resistance and relapse following treatment with vemurafenib are major problems, and the average tumor-free survival time after treatment has remained less than one year [5]. Furthermore, a number of countries have excluded this drug from subsidy within their health care programs due to a high cost/benefit ratio. Additional compounds and antibodies for targeted, immuno- as well as combination therapy have been and are currently being developed such as the BRAF^V600mut^ inhibitor dabrafenib [6], mitogen-activated protein kinase kinase (MEK) inhibitors trametinib [7] and cobimetinib [8], the anti-cytotoxic T-lymphocyte-associated protein 4 (CTLA-4) antibody ipilimumab [9] as well as anti-programmed cell death protein 1 (PD-1) antibodies pembrolizumab [10] and nivolumab [11]. However, despite promising results being achieved with these agents, lack of responsiveness and relapse are still major problems (reviewed in [12, 13]).

Differing from a large number of solid tumors, the tumor suppressor p53 is rarely mutated in melanoma [14-18]. Instead, its function is thought to be abrogated by other mechanisms, such as overexpression of its negative regulators murine double minute 2 (mdm2) or mdm4 (mdmx) [19-21]. During the past decade, several classes of non-genotoxic compounds that can reactivate wild-type (wt) p53 by inhibiting its interaction with mdm2 and/or mdm4 have been described (reviewed in [22]). The most widely tested of these is nutlin-3 [23], a derivative of which, RG7112 (RO5045337), has been tested in phase I clinical trials [24, 25]. However, nutlin-3 can lead to both cytotoxic and cytostatic effects [23, 26-28]. Whilst cytotoxic effects are desirable, cell cycle arrest can lead to the recovery of cell populations following drug removal [28], and may result in relapse. Thus, finding other non-genotoxic p53 activators that specifically lead to death in tumor cells is desirable. Therefore, we performed a melanoma cell-based screen to identify compounds that can activate p53, and subsequently studied the capability of selected compounds to induce cytotoxicity rather than cell cycle arrest, in this cell type.

In the present study we describe characteristics of the non-genotoxic small molecule MJ25 ((2-{[2-(1,3-benzothiazol-2-ylsulfonyl)ethyl]thio}-1,3-benzoxazole), PubChem compound ID 1319216), named “MJ25” for screen compound 25. MJ25 was an active compound in our screen for p53 reactivators, albeit with low potency. However, as MJ25 was also independently identified as a hit compound in a recent quantitative high-throughput screen for inhibitors of the selenoprotein thioredoxin reductase 1 (TrxR1), an enzyme of major importance in cellular redox control [29-31], it was selected for further characterization. The results of that screen, based upon a previously described assay [32], have recently been deposited in PubChem (BioAssay number: 588453; W. Stafford et al., manuscript in preparation). The combined activities of MJ25 to activate p53 and simultaneously inhibit TrxR1 were interesting features from a potential therapeutic perspective and resembled effects previously noted for compounds such as RITA [33] and PRIMA-1^MET^ [34]. We therefore wished to study the cytotoxic properties of MJ25 in melanoma cells.

TrxR1 is a cytosolic antioxidant protein with the main function of keeping the active site disulfide/dithiol motif of thioredoxin 1 (Trx1) in a reduced state. TrxR1 thereby supports a wide range of Trx1-dependent cellular pathways, from providing protection against excessive levels of reactive oxygen species (ROS) [29, 30, 35] to modulating several levels of redox regulation and cell viability [31]. To perform its catalytic function, TrxR1 needs to be present in a reduced state. TrxR1 reduction is a multistep process initiated by nicotinamide adenine dinucleotide phosphate (NADPH) reducing a flavin adenine dinucleotide (FAD) moiety. Subsequently, FADH_2_ reduces the redox-active disulfide motif -CVNVGC- in the N-terminus of the same subunit of the homodimer, resulting in a dithiol motif. Finally, this dithiol reduces the selenium-sulfide motif formed by the cysteine (Cys) and selenocysteine (Sec) residues in the -GCUG sequence present in the C-terminus of the other subunit. This selenolthiol can, in turn, reduce most of the enzyme's substrates including Trx1. The C-terminal selenolthiol motif of the NADPH-reduced enzyme is easily accessible to substrates and is considered to be the major active site of the enzyme [36-38]. However, substrates like juglone (5-hydroxy-1,4-naphthoquinone, walnut toxin) can be reduced by the N-terminal FAD/–CVNVGC- motif in a Sec-independent manner [39].

The growth-stimulatory and anti-apoptotic activities of TrxR1, as well as its observed upregulation in a number of tumor types, suggests that inhibition of TrxR1 may result in tumor growth inhibition [40]. Indeed, various compounds with Trx- or TrxR1-inhibitory activities have been described as potential anti-cancer agents [35, 40-42]. Auranofin (Ridaura) is a potent inhibitor of TrxR1 [43-50] and approved for the treatment of rheumatoid arthritis, and is currently being tested for potential anti-cancer activity in a small number of clinical trials (http://www.clinicaltrials.gov). However, its activity against malignant melanoma is not currently being evaluated in those trials. Here we found that both MJ25 and auranofin inhibit TrxR1 and are cytotoxic towards melanoma cells. These compounds also display similarities regarding p53 protein induction and BRAF^V600mut^ dependence. Our data suggest that targeting TrxR1 may indeed be a possible therapeutic strategy for the treatment of melanoma.

## RESULTS

### MJ25 and its p53 related properties

**Figure 1:**
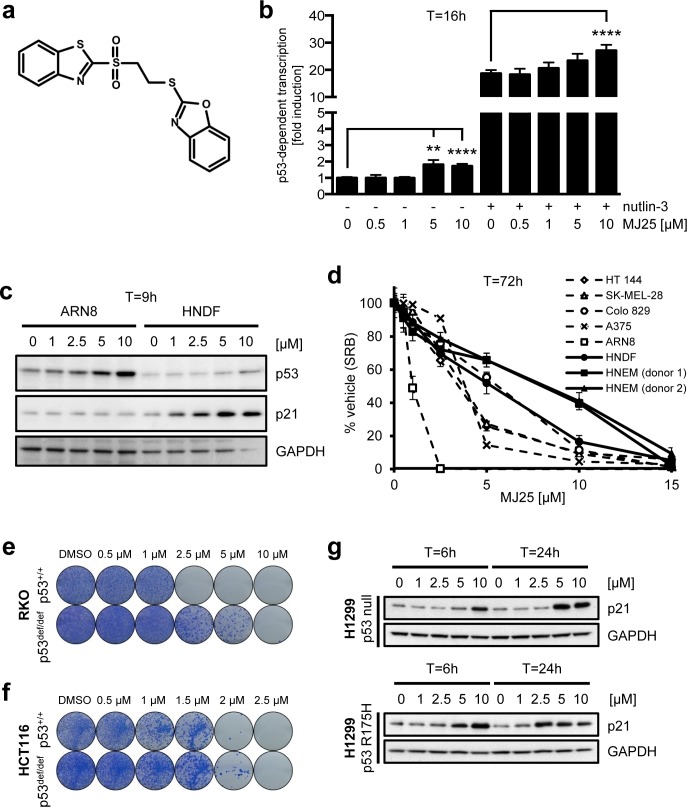
MJ25 activates p53 and eradicates melanoma cells in culture **a.** Chemical structure of MJ25. **b.** ARN8 cells, which express wt p53 and have been stably transfected with a p53-dependent β-galactosidase expression vector (RGC-ΔFos-LacZ reporter plasmid), were co-treated with vehicle (−) (DMSO) or nutlin-3 [2 μM] (+) and vehicle (0) (DMSO) or MJ25 at the indicated concentrations for 16 hours. p53-dependent transcription was assessed by measurement of β-galactosidase activity, taking protein levels into consideration. Error bars represent standard deviation. **, *p* < 0.01; ****, *p* < 0.001 (unpaired one-tailed Student's *t*-test; *n* = 4). **c.** ARN8 cells and human normal dermal fibroblasts (HNDFs) were treated with MJ25 at increasing concentrations for 9 hours. Protein levels were determined by Western blotting. GAPDH served as loading control. **d.** Cell growth and viability were measured in a number of melanoma cell lines, HNDFs and human normal epithelial melanocytes (HNEMs) by sulforhodamine B (SRB) assay after treatment with MJ25 at the indicated concentrations for 72 hours. Error bars represent standard deviation. (e and f) The effect of MJ25 on cell viability and colony-forming capacity was studied in **e.** RKO p53^+/+^ and p53^def/def^ cells as well as **f.** HCT116 p53^+/+^ and p53^def/def^ cells. **g.** H1299 cells (p53 null; top panel) and H1299 cells stably transfected with mutant p53 (R175H; bottom panel) were treated with MJ25 at the indicated concentrations for each 6 or 24 hours, respectively. p21 levels were determined by WB. GAPDH was used as loading control.

To identify small molecules that activate wt p53 in melanoma we performed a screen in ARN8 cells expressing β-galactosidase under the control of a p53-dependent promoter. MJ25 (Figure 1a) was identified as an active compound in this screen, displaying modest p53 activation. As it was also identified as a TrxR1 inhibitor with an IC_50_ of 1.122 μM (PubChem BioAssay number: 588453; http://pubchem.ncbi.nlm.nih.gov), it was selected for further analysis. MJ25 was subsequently repurchased and its activation of p53-dependent transcription confirmed in ARN8 cells as well as in the T22 cell line, a murine prostate-derived cell line stably transfected with the same β-galactosidase reporter gene as ARN8 cells (data not shown) [51]. We also found that MJ25 further increased the p53 transcription factor activity in ARN8 cells when used in combination with nutlin-3, a well-described specific p53 activator (Figure 1b) [23], suggesting that the two compounds have different mechanisms of action. Interestingly, MJ25 induced p53 protein levels in ARN8 cells without increasing p21 levels, whilst mainly inducing p21 and not p53 in human normal dermal fibroblasts (HNDFs) (Figure 1c). We found that MJ25 reduced the growth of various melanoma cell lines after 72 hours of treatment, whilst being less toxic to both HNDFs and human normal epithelial melanocytes (HNEMs) (Figure 1d). Supporting the observation that p53 activation by MJ25 was modest, its ability to kill tumor cells was weakly dependent on the presence of full-length-p53. A slight decrease in sensitivity was observed in RKO and HCT116 cell lines deficient in full-length p53 (p53^def/def^) compared with their p53^+/+^ isogenic counterparts (Figure 1e and 1f). The increased levels of p21 levels in HNDFs and not ARN8 cells (Figure 1c) suggested that the ability of MJ25 to induce this protein was enhanced in the absence of activated p53. In support of this finding, p21 was also strongly induced in p53-null H1299 cells as well as in H1299 cells stably transfected with mutant p53 (Figure 1g).

**Figure 2:**
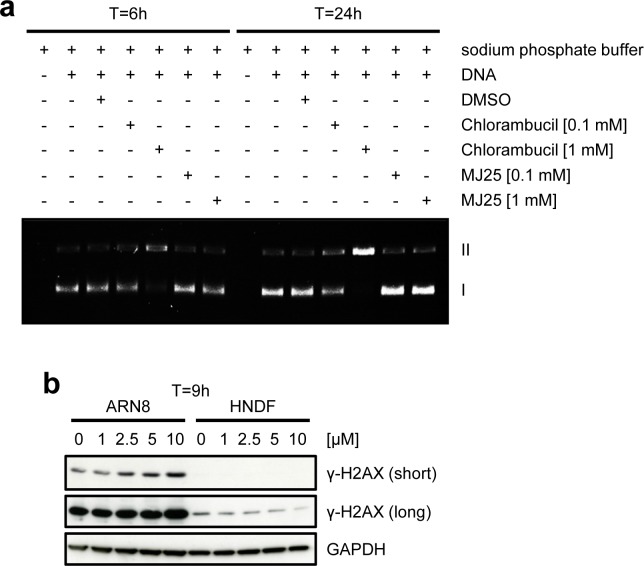
MJ25 appears to be non-genotoxic **a.** MJ25's DNA alkylating capacity was assessed in an *in vitro* DNA alkylation assay. Form I (lower band) represents supercoiled (unaffected) plasmids and form II (upper band) open circular plasmids, which appear upon DNA alkylation. **b.** ARN8 cells and HNDFs were treated with MJ25 at various concentrations for 9 hours. Changes in levels of γ-H2AX were determined by Western blotting. GAPDH served as loading control.

p53 activation suggested that MJ25 may act as a DNA damaging agent, and the presence of a sulfone group in this compound suggested that it may do so by DNA mono-alkylation. However, such activity could not be detected in an assay for DNA alkylation (Figure 2a). We also determined whether MJ25 increased the levels of γ-H2AX, which occurs in response to double-strand breaks (DSBs) [52] and is often used as an indicator of possible genotoxicity. MJ25 did not induce γ-H2AX in HNDFs within 9 hours of exposure (Figure 2b) nor at later times (data not shown). γ-H2AX levels were slightly increased in ARN8 cells at concentrations of MJ25 that lead to cytotoxicity in these cells (Figures 1d and 2b). Cell death driven DNA fragmentation, which can also result in increased levels of γ-H2AX [53], may account for this result.

### The dependency of MJ25's cytotoxicity on mutant BRAF

**Figure 3:**
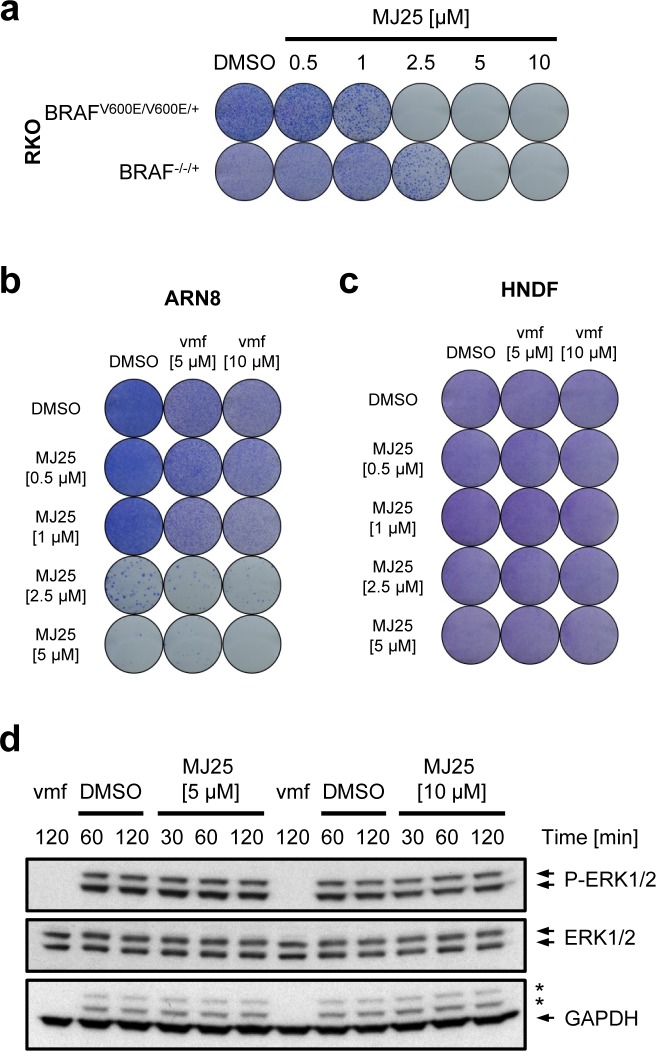
MJ25's cytotoxic effect is enhanced by mutant BRAF **a.** RKO BRAF^V600E/V600E/+^ and BRAF^−/−/+^ cells were treated for 72 hours with MJ25 as indicated, and cell viability and clonogenic capacity were determined. (b and c) The effect of MJ25 either alone or in combination with vemurafenib (vmf) on cell viability and clonogenic capacity was determined in **b.** ARN8 cells and **c.** HNDFs. DMSO served as vehicle control. **d.** ARN8 cells were treated with vemurafenib (vmf) [5 μM], DMSO or MJ25, respectively, at the indicated concentrations for the indicated periods of time. Changes in protein levels were determined by Western blotting. Stars indicate bands representing phospho-ERK subunits that remained when re-using the membrane for blotting against GAPDH.

All of the melanoma cells tested here harbor a V600E point mutation in BRAF, a mutation that occurs in approximately 50% of patients suffering from melanoma [4]. We therefore tested if the cytotoxic effects of MJ25 were dependent on a constitutively active BRAF pathway. Both the ARN8 and RKO cell line express BRAF^V600E^ [54, 55], which drives their proliferation and survival [56-58]. As shown in Figure 3a, MJ25 was slightly more potent at killing tumor cells expressing BRAF^V600E^ than isogenic cells lacking this mutant protein. Notably, MJ25 was able to kill ARN8 cells that were co-treated with vemurafenib, the first inhibitor of BRAF^V600E^ clinically approved for the treatment of unresectable or metastatic melanoma [3, 4] (Figure 3b). MJ25 was furthermore able to induce cell death in cells that were largely insensitive to vemurafenib, achieving almost total cell eradication both as a single agent and when combined with vemurafenib (Figure 3b). In contrast, neither single nor combined treatment affected the clonogenic potential of HNDFs (Figure 3c).

Unlike vemurafenib, MJ25 did not inhibit phosphorylation of extracellular signal-regulated kinases 1 and 2 (ERK1 and ERK2), which are downstream targets of BRAF and indicative of BRAF activation (Figure 3d). Thus, MJ25 is mechanistically different from vemurafenib and its effects on the BRAF pathway are likely to be indirect.

### Inhibition of thioredoxin reductase 1 by MJ25 in comparison to auranofin

**Figure 4:**
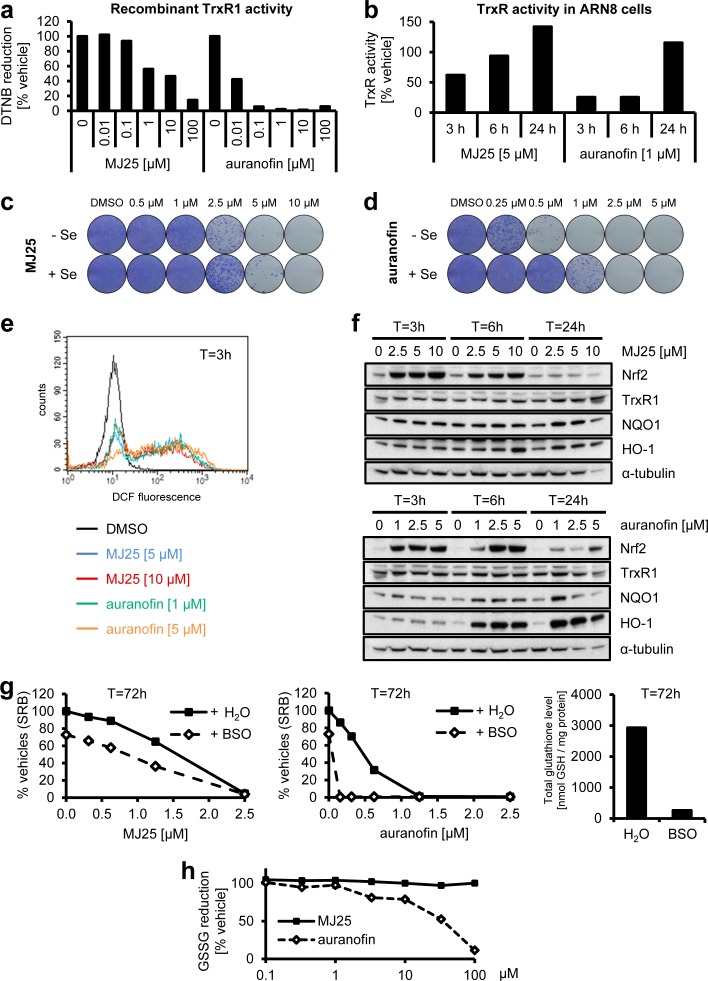
MJ25 is an inhibitor of thioredoxin reductase 1 (TrxR1) **a.** The capability of MJ25 and auranofin to inhibit recombinant, rat-derived TrxR1 *in vitro* was measured by an NADPH dependent 5,5′-dithiobis-[2-nitrobenzoic acid] (DTNB) assay. **b.** ARN8 cells were treated with MJ25, auranofin or DMSO, respectively, for the indicated periods of time. TrxR1 inhibition was subsequently assessed in cell lysates by an NADPH and Trx dependent insulin reduction endpoint assay, measuring thiol formation using DTNB. Ratios between MJ25 and DMSO as well as auranofin and DMSO were determined for each point in time. (c and d) ARN8 cells were treated with **c.** MJ25 or **d.** auranofin, while in each half of the samples growth media were supplemented with sodium selenite [75 nM] three days prior to seeding as well as during seeding and treatment for 72 hours. Cell viability and clonogenic capacity were determined. **e.** ROS levels were determined in ARN8 cells 3 hours after the indicated treatment by measuring fluorescence of 2′,7′-dichlorofluorescein (DCF). **f.** Induction of anti-oxidative proteins by MJ25 and auranofin was investigated in ARN8 cells at the indicated points in time by Western blotting. DMSO served as vehicle control (0 μM). **g.** ARN8 cells were pre-treated with L-buthionine sulfoximine (BSO) or vehicle (H_2_O) for 72 hours, upon which cells were re-plated in BSO- and vehicle-free growth medium. Cell viability was assessed by SRB assay after 72 hours in the presence of vehicle (DMSO), MJ25 (left panel) or auranofin (middle panel), respectively. Intracellular glutathione (GSH) levels were determined 72 hours after BSO / vehicle treatment (right panel). **h.** Inhibition of yeast-derived glutathione reductase by MJ25 and auranofin was determined *in vitro* by measurement of glutathione disulfide (GSSG) reduction.

In addition to reactivating wild-type p53, MJ25 was independently identified as an inhibitor of TrxR1 (see above). Therefore, we also confirmed that repurchased MJ25 inhibited recombinant TrxR1 (Figure 4a) and then tested whether MJ25 inhibited TrxR1 in ARN8 cells (Figure 4b). When analyzing cells treated with 5 μM MJ25 we found that the compound transiently inhibited TrxR activity in cell lysates at 3 hours of treatment, with a subsequent rebound effect at later time points (Figure 4b). This effect was also seen with auranofin, a well-described and very potent TrxR inhibitor (Figure 4a and 4b) [43-50]. Such a rebound effect is likely to be nuclear factor (erythroid-derived 2)-like 2 (Nrf2) dependent [59]. This is supported by the finding that both MJ25 and auranofin strongly induce this factor (Figure 4f, see also below).

To further probe if TrxR1 targeting may be a component of MJ25's mechanism of action, we increased the expression of selenoproteins by supplementing the growth medium with sodium selenite [60]. The effects of both MJ25 and auranofin on colony-forming capability were clearly dampened by trace amounts of sodium selenite (Figure 4c and 4d). Auranofin was more affected by selenium supplementation than MJ25, suggesting that auranofin is more selenoprotein dependent than MJ25 (Figure 4a and 4b).

Since TrxR1 plays a key role in the cellular antioxidant defense [29, 30, 35], we next investigated whether MJ25 or auranofin induces changes in ROS levels or upregulates antioxidant pathways. Both MJ25 and auranofin might induce ROS formation, as suggested by the observed increase in 2′,7′-dichlorofluorescein (DCF) levels (Figure 4e). Treatment of ARN8 cells with either compound also led to a dose- and time-dependent induction of nuclear factor Nrf2, a key factor in cellular antioxidant responses [61, 62], and, to a lesser extent, increases in the Nrf2 transcriptional targets NAD(P)H:quinone oxidoreductase 1 (NQO1), heme oxygenase-1 (HO-1), and TrxR1 itself (Figure 4f). This induction of TrxR1 expression may indeed explain the transient loss of TrxR1 inhibition by MJ25 and auranofin as well as the fact that TrxR1 activity was higher 24 hours post-treatment compared to its starting activity (i.e. >100%) (Figure 4b). Of note, the effects of MJ25 on induction of Nrf2 and its target genes were less persistent over time than those of auranofin, and the latter caused a significant level of cell death at the highest dose after 24 hours of treatment (Figure 4f).

To further study the dependence of MJ25-induced cytotoxicity on TrxR1 and Trx1-dependent pathways, ARN8 cells were pre-treated with L-buthionine sulfoximine (BSO) to deplete intracellular glutathione (GSH) levels by inhibiting glutathione synthesis (Figure 4g, right panel) [63]. Cell growth decreased approximately 30% upon BSO treatment alone. Co-treatment with auranofin led to complete eradication of cell populations, which is in line with the assumption that simultaneous inhibition of the glutathione and Trx systems will lead to enhanced cytotoxicity [64]. The cytotoxicity of MJ25 was less affected by BSO pre-treatment (Figure 4g), which correlates well with its lower TrxR1-inhibitory capacity in cell culture compared with auranofin. Of note, glutathione reductase (GR), the enzyme that converts glutathione disulfide (GSSG) to its reduced form (GSH), is highly homologous to TrxR1, in particular in its catalytically active site, and both enzymes are NADPH- and FAD-dependent [65, 66]. Therefore, we tested if MJ25 is able to inhibit GR *in vitro*. The data presented in Figure 4h suggest that this is not the case, as MJ25 did not inhibit the enzyme at any dose tested. Auranofin was tested for comparison, and inhibition occurred only at concentrations exceeding those required for having effects on cells.

### MJ25 and auranofin are irreversible inhibitors of TrxR1 and inhibit Sec-dependent activities of the enzyme

**Figure 5:**
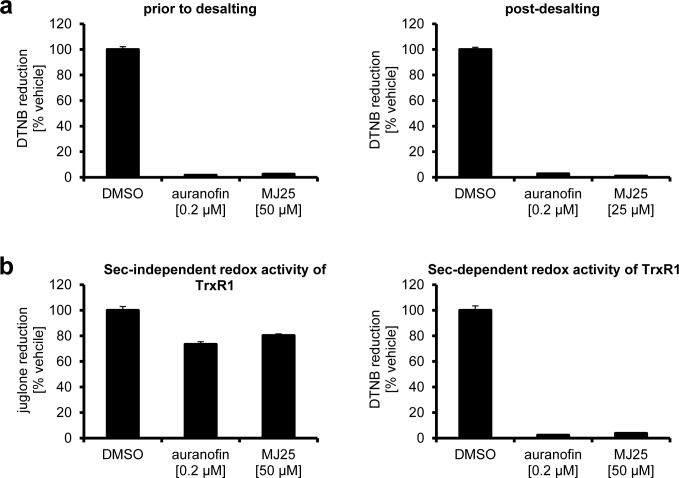
Inhibition of TrxR1 by MJ25 and auranofin is irreversible and likely occurs at its Sec-dependent active site **a.** NADPH-reduced TrxR1 was incubated with compounds as indicated, and Sec-dependent enzyme activity was subsequently measured by an NADPH dependent DTNB reduction assay (left panel). Reversibility of inhibition was investigated by desalting of the enzyme and subsequent determination of enzyme activity by the NADPH dependent DTNB assay (right panel). **b.** Sec-independent activity of TrxR1 was determined with an aliquot of TrxR1 incubated with compounds as indicated, followed by measurement of NADPH dependent juglone reduction (left panel). To ensure complete inhibition of the Sec-dependent active site, DTNB activity was tested with the same master mixes of enzyme and compounds (right panel).

Electrophilic compounds can easily target the selenocysteine containing, redox active site of TrxR1 which is located at the C-terminus of the enzyme. Small molecule inhibition of TrxR1 at the C-terminus is often irreversible and, depending on the inhibitor, may form Selenium compromised Thioredoxin Reductase-derived Apoptotic Proteins (SecTRAPs) [67, 68]. SecTRAPs gain pro-oxidant activities directly in contrast to uninhibited TrxR1 functions via sustained NADPH consumption through the N-terminal dithiol motif, actively producing ROS and inducing cell death despite the derivatized protein's inability to reduce the traditional substrates of TrxR1, such as Trx1 [39]. This unique SecTRAP activity can be observed through reduction of juglone [39]. Auranofin, an electrophilic compound, was previously shown to irreversibly inhibit TrxR1 and induce SecTRAP formation [69, 70]. Here, we found that also MJ25 was able to irreversibly inhibit TrxR1 (Figure 5a). MJ25, like auranofin, also sustained SecTRAPs-like redox cycling activity with juglone as a substrate after complete inhibition of the C-terminal active site (Figure 5b).

### Effects of auranofin on cell viability, p53 activity and levels of γ-H2AX

**Figure 6:**
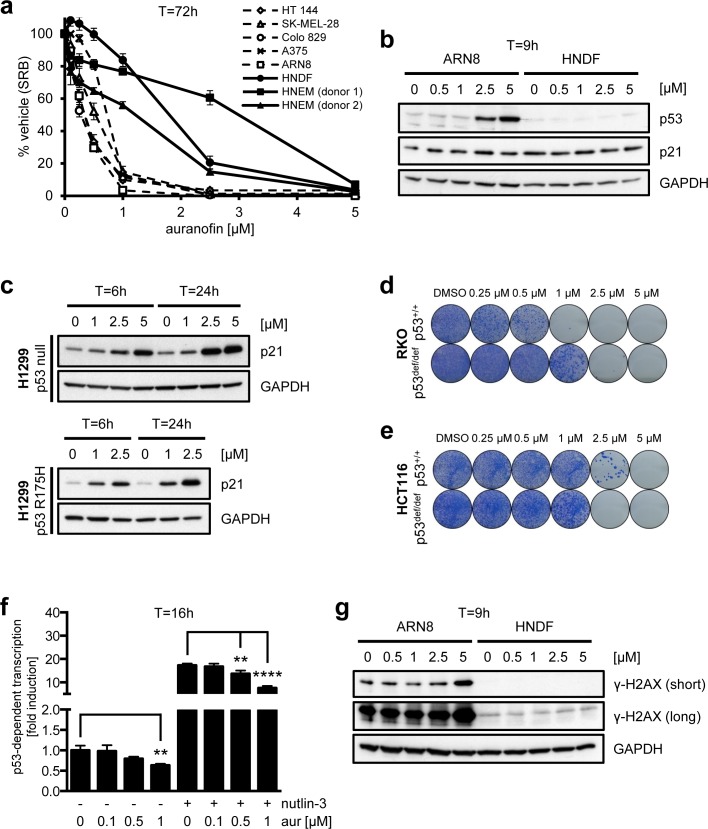
Effects of auranofin on melanoma cell viability and p53 **a.** A number of melanoma cell lines as well as HNDFs and HNEMs were treated with auranofin at various concentrations for 72 hours. Cell growth and viability were measured by SRB assay. Data are representative of four independent experiments; error bars represent standard deviation. **b.** ARN8 cells and HNDFs were treated with auranofin at increasing concentrations for 9 hours. Changes in protein levels were determined by Western blotting. GAPDH was used as loading control. **c.** H1299 cells (p53 null; top panel) and H1299 cells stably transfected with mutant p53 (R175H; bottom panel) were treated with auranofin at the indicated concentrations for each 6 or 24 hours, respectively. p21 levels were determined by Western blotting. GAPDH served as loading control. (d and e) The effect of MJ25 on cell viability and colony-forming capacity was studied in **d.** RKO p53^+/+^ and p53^def/def^ as well as **e.** HCT116 p53^+/+^ and p53^def/def^ cells. **f.** ARN8 cells were treated with DMSO (−) or nutlin-3 [2 μM] (+) and DMSO (0) or auranofin (aur) at the indicated concentrations for 16 hours. p53-dependent transcription was assessed by measurement of β-galactosidase activity under consideration of protein levels. Error bars represent standard deviation. **, *p* < 0.01; ****, *p* < 0.001 (unpaired two-tailed Student's *t*-test; *n* = 4). **g.** ARN8 cells and HNDFs were treated and analyzed as in **b.**.

Auranofin has previously been tested in various tumor cell lines in connection with the Developmental Therapeutics Program (DTP) by the National Cancer Institute (NCI) at the National Institutes of Health (NIH) (Cancer Chemotherapy National Service Center (NSC) number: 321521) (http://dtp.nci.nih.gov/index.html) [71]. Analyzing those data, it is evident that auranofin can inhibit cell growth in the majority of cell lines, with effective doses up to 3 μM (Supplemental Figure S1). Strikingly, the GI_50_ value (concentration of drug required for 50% growth inhibition) was 0.5 μM or less in all of the melanoma cell lines tested. We therefore wished to further analyze the effects of auranofin on melanoma cells. First we confirmed its growth inhibitory activity towards melanoma cells in several cell lines (Figure 6a). In a manner similar to MJ25, we also found that auranofin displayed milder effects on both HNDFs and HNEMs (Figure 6a). Furthermore, at higher concentrations auranofin was also able to increase p53 protein levels in ARN8 cells (Figure 6b), and potent induction of p21 was observed in the absence of wt p53 (Figure 6c). However, unlike MJ25, auranofin did not induce p21 in HNDFs (Figure 6b). Interestingly, the manner by which the p53 status influenced the cytotoxicity of auranofin was cell line specific. The presence of wt p53 affected the clonogenic potential of RKO cells (Figure 6d), but slightly protected HCT116 cells from the drug (Figure 6e). Strikingly, auranofin inhibited rather than stimulated the transcriptional activity of p53, which was an effect most clearly illustrated in combination with nutlin-3 (Figure 6f). This finding indicates a major difference in the cellular responses to auranofin and MJ25. Auranofin also slightly induced the expression of γ-H2AX in ARN8 cells, but not in HNDFs (Figure 6g), which again may be the consequence of DNA fragmentation occurring during cell death.

### The cytotoxic effect of auranofin is partially BRAF^V600mut^-dependent

**Figure 7:**
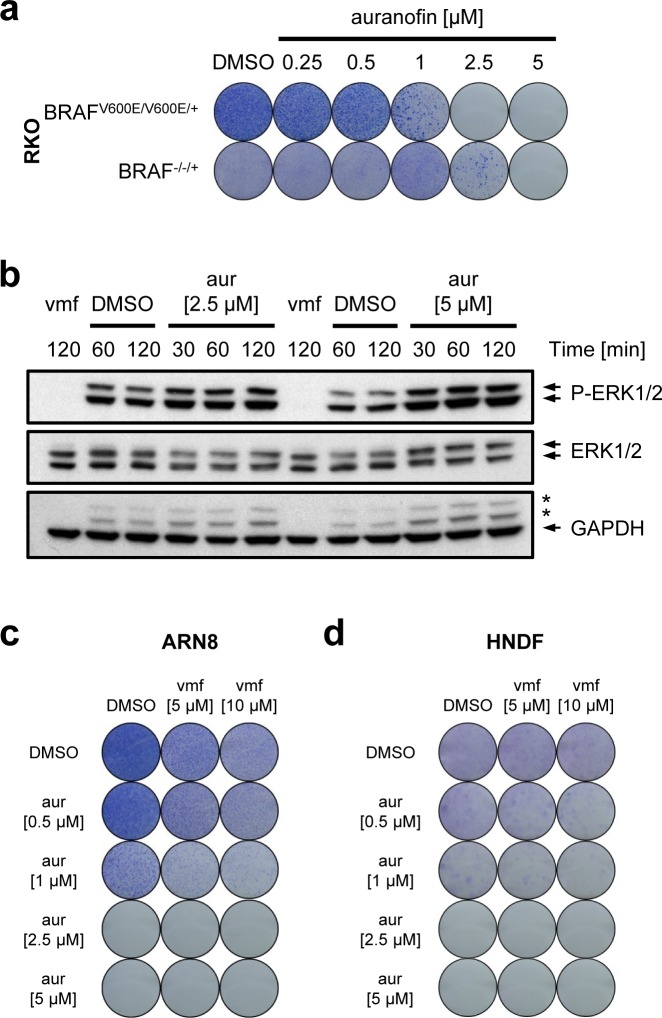
Auranofin's cytotoxic effect in relation to mutant BRAF **a.** RKO BRAF^V600E/V600E/+^ and BRAF^−/−/+^ cells were treated for 72 hours with auranofin as indicated, and cell viability and clonogenic capacity were determined. **b.** ARN8 cells were treated with vemurafenib (vmf) [5 μM], DMSO or auranofin, respectively, at the indicated concentrations for the indicated periods of time. Changes in protein levels were determined by Western blotting. GAPDH was used as loading control. Stars indicate bands representing phospho-ERK subunits that remained when re-using the membrane for blotting against GAPDH. (c and d) ARN8 cells **c.** and HNDFs **d.** were treated with auranofin at increasing concentrations in combination with DMSO or vemurafenib (vmf) as indicated. Viability and clonogenic potential were assessed.

As seen for MJ25 (Figure 3a), auranofin was more efficient at killing cells expressing mutant BRAF (BRAF^V600E/V600E/+^) than an isogenic cell line in which mutant BRAF had been knocked out (BRAF^−/−/+^) (Figure 7a) [55]. Furthermore, auranofin increased the levels of phosphorylated ERK1/2 (Figure 7b), indicating that the drug did not directly inhibit BRAF. In addition, auranofin was able to eradicate the entire melanoma cell population, including cells that were largely insensitive to vemurafenib (Figure 7c). Notably, however, auranofin affected the clonogenic potential of HNDFs as strongly as that of ARN8 cells, an effect that was seen both in the presence and absence of vemurafenib (Figure 7c and 7d).

## DISCUSSION

Here we demonstrate that MJ25, an inhibitor of TrxR1 and weak inducer of p53 activity, can efficiently kill a number of melanoma cell lines, whilst having milder effects on HNDFs and HNEMs in culture. We also found auranofin, a more potent TrxR1 inhibitor, to have similar effects on melanoma cells. However, auranofin and MJ25 display differences with regard to their efficacy, selectivity between melanoma cells and normal cells, dependence upon the glutathione system, as well as their effects on signaling events downstream of p53.

Both MJ25 and auranofin were found to inhibit TrxR1 irreversibly. It has been suggested earlier that auranofin can derivatize the Sec residue of TrxR1 due to its electrophilic nature [69], which should account for the observed irreversible inhibition. MJ25 carries a sulfone group, which may be the site where it reacts with its target. However, this remains to be shown. Interestingly, our data suggest that MJ25 neither alkylates DNA nor inhibits the dithiol/disulfide-containing GR, suggesting that Sec may be the preferential target.

Whilst TrxR1 helps to prevent cellular damage caused by ROS [29, 35], inhibition of TrxR1 can itself lead to ROS production by the conversion of the enzyme to a pro-oxidant NADPH oxidase [68, 72-75]. Increased ROS levels have been shown to induce p53 expression [76], which may explain the increase in p53 protein levels upon treatment with either MJ25 or auranofin. However, only MJ25 was able to induce p53's transcriptional activity, whereas auranofin inhibited the activity of p53, an effect that was even more obvious in the presence of nutlin-3. An earlier study suggests that treatment with auranofin may lead to a conformational change in p53, resulting in the inability of the latter to bind to its consensus sequence, and that this conformational change might be caused by inhibition of the Trx pathway [77]. Since MJ25 is less potent towards TrxR1 inhibition, only a fraction of the p53 protein molecules may undergo a conformational change. This, in turn, may also explain why the transcriptional activity of p53 is not increased proportionally to its protein levels upon MJ25 treatment. Conversely, we found that the p53 status could affect the cytotoxicity of auranofin on tumor cells and their colony-forming capabilities, but in a cell line dependent manner. In contrast, the cytotoxicity of MJ25 was similar in two sets of cell lines (HCT116 and RKO), with MJ25 being more effective in cells expressing wt p53 than in the respective isogenic p53-deficient cell lines. The reasons for these qualitative differences in effects between MJ25 and auranofin are not clear, but could relate to different off-target or compartmentalization effects. For example, it is well known that auranofin is accumulated in mitochondria and first exerts its effects there [45, 48, 49, 78, 79], while differential organellar effects of MJ25 are not yet known. However, the effects of TrxR inhibition, p53 protein induction, ROS production, Nrf2 induction and preferential cancer cell cytotoxicity are features evidently shared between the two compounds. Interestingly, another anticancer drug candidate, RITA, displays a similar profile of effects with TrxR1 inhibition, ROS induction and p53 activation [33], suggesting the potential of a general anticancer principle. Indeed, non-p53 related effects are difficult to exclude [80].

We also found here that the cytotoxicity caused by MJ25 and auranofin was slightly BRAF-dependent, even though the compounds were not direct inhibitors of the mutant BRAF kinase. Interestingly, BRAF^V600E^ has previously been shown to induce Nrf2 [81], and phosphorylation of the BRAF downstream targets ERK1/2 were found here to be induced by treatment of cells with auranofin, suggesting an intricate web of signaling with regard to BRAF, p53, Nrf2 and TrxR1 status. Tumor cells in which the BRAF pathway is constitutively active, due to the presence of mutant BRAF, generally have a high rate of proliferation. Therefore, they need to compensate for the high amounts of ROS produced during frequent rounds of cell division and a high rate of metabolism. Thus, disruption of ROS regulation in melanoma cells, in which the levels of ROS might be quite high already, might explain why they respond more strongly by undergoing cell death than their isogenic counterparts in which the BRAF pathway is not overactive. It should be noted, however, that TrxR1 inhibition typically induces Nrf2 [59], as also seen here, suggesting that sub-cytotoxic doses of auranofin or MJ25 may potentially promote proliferation and cell viability. Thus, both concentration- and time-dependency should likely be important for a final outcome using auranofin, MJ25 or other drugs with similar profiles.

The increase in the levels of phosphorylated ERK1/2 upon treatment with auranofin may be attributed to increased ROS levels, as it has been reported previously that ROS can induce higher levels of phosphorylated ERK1/2 [82]. Furthermore, our data confirm a previous study, in which auranofin was shown to induce phosphorylation of ERK1/2 through the generation of ROS [82]. In contrast, MJ25 was not able to induce phosphorylation of ERK1/2 despite its ability to induce ROS. However, MJ25 is less potent than auranofin with regard to TrxR1 inhibition as well as induction of proteins involved in the anti-oxidant response, suggesting that additional pathways modulate phosphorylation of ERK1/2 in different manners when comparing the effects of MJ25 to those of auranofin.

We have found that a significant fraction of ARN8 cells undergo arrest in G1-phase of the cell cycle even at very high doses of vemurafenib, and that these cells can recover as soon as the drug has been removed (manuscript in preparation). This may be related to resistance to vemurafenib, which is of major concern, even though new treatment schedules may reduce or overcome this problem [83]. The data presented here confirm that even high doses of vemurafenib could not lead to complete eradication of melanoma cell populations. Importantly, auranofin led to complete cell killing without any traces of cells recovering from treatment. Melanoma cells are very sensitive to changes in ROS, which may be part of the explanation of these effects. Induction of ROS has indeed been proposed as an effective way of killing melanoma cells, especially of those that are resistant to BRAF inhibitors like vemurafenib [84]. Because both MJ25 and auranofin were here found to have milder effects in normal cells than in melanoma cells with regard to cell viability, this suggests a certain cancer cell selectivity, even though the clonogenic potential of HNDFs was significantly affected by auranofin. However, the FDA approval of auranofin (Ridaura) as an anti-rheumatic agent and its use in current clinical trials for cancer treatment show that the drug can be tolerated by patients.

The molecular mechanisms for the differences between MJ25 and auranofin require further investigation. Importantly, the potency of MJ25 was not significantly improved by GSH depletion, which was in stark contrast to that of auranofin. This suggests that MJ25 and/or auranofin may have different other targets beyond TrxR1, the inhibition of which may contribute to death of melanoma cells. A search in PubChem revealed that aldehyde dehydrogenase (ALDH) 1A1 may be inhibited by MJ25 at sub-micromolar concentrations (BioAssay number: 1030). The ALDH superfamily consists of 19 genes in humans, and some members have been shown to play a role in cancer stemness [85]. In particular, ALDHs may be involved in cancer stemness in melanomas [86], and ALDH1A1 has been identified as one of the key ALDH isozymes involved in this process [87]. Thus, we investigated if MJ25 kills melanoma cells via inhibition of ALDH1A1 by comparing the effects of MJ25 on p53 activation, cell growth and clonogenic potential to those of disulfiram, which is an inhibitor of ALDH1 and ALDH2 [88] and approved for the treatment of alcoholism [89]. However, since disulfiram neither activated the transcriptional activity of p53 nor showed any selectivity in clonogenic assays comparing ARN8 cells with HNDFs (data not shown), we concluded that inhibition of ALDH, or in particular ALDH1A1, cannot be the key mechanism for selective cytotoxic effects of MJ25. Thus, it remains to be determined which other target(s) or mechanism(s) of MJ25 elicit its effects in a manner independent of the GSH status of cells.

Due to its poor solubility, MJ25 has not yet been tested *in vivo*. Chemical modification of the compound to increase its solubility would be a preferable next step in development of the compound as a potential anticancer drug candidate, combined with structure-activity relationship (SAR) studies. In contrast, auranofin has already been clinically approved for the treatment of rheumatoid arthritis. With clinical trials testing auranofin against a number of different types of cancer currently being performed (http://www.clinicaltrials.gov) but melanoma not being amongst them, we suggest that auranofin might be a good drug candidate also for the treatment of melanoma.

## MATERIALS AND METHODS

### Reagents and compounds

Acetic acid (no. 33209), acetone (no. 32201), agarose (no. A9539), auranofin (no. A6733), bovine serum albumin (BSA; no. A9647), dimethyl sulfoxide (DMSO; no. D8418), BSO (no. B2515), chlorambucil (no. C0253), 2′,7′-dichlorofluorescein diacetate (DCF-DA; no. 35845), ethidium bromide (no. E1385), Giemsa solution (no. 48900), insulin (no. 15500), juglone (no. H47003), methanol (no. 3221N), N,N′-dimethylethylenediamine (DMEDA; no. D157805), nutlin-3 (no. N6287), Ponceau S (no. P3504), sodium phosphate monobasic (no. S5011), sodium phosphate dibasic (no. S7907), sodium selenite (no. S1382), 5-sulfoalicylic acid (SSA; no. 52130), sulforhodamine B (SRB; no. S9012), trichloroacetic acid (TCA; no. T6399), tris base (no. T1503) and Tween 20 (no. P2287) were obtained from Sigma-Aldrich (Heidelberg, Germany). Glutathione reductase derived from baker's yeast (*Saccharomyces cerevisiae*) (no. 359960) and Tris-HCl (no. 648313) were purchased from Calbiochem / Merck (Darmstadt, Germany). 5,5′-dithiobis-[2-nitrobenzoic acid] (DTNB; no. 422593K) and HCl (no. 2611.5000) were from VWR. Chlorophenol red-β-D-galactopyranoside (CPRG; no. 884308) was purchased from Roche (Mannheim, Germany). MJ25 (ChemBridge ID: 7617239) was obtained from ChemBridge (San Diego, CA, USA). Vemurafenib (no. S1267) was purchased from Selleckchem (Houston, TX, USA). Ethylenediaminetetraacetic acid (EDTA; no. 1.08417.1000) was from Merck (Darmstadt, Germany) and Sigma-Aldrich (no. ED2SS), and supercoiled pHOT1 DNA (no. TG2030) from Topogen (Port Orange, FL, USA). Reduced glutathione (GSH; no. A2084.0005), oxidized glutathione (GSSG; no. G4376), and NADPH (no. A1395.0500) were purchased from AppliChem (Kongens Lyngby, Denmark). DMSO was used as vehicle for MJ25, auranofin and vemurafenib. Compound stock solutions were stored at −20°C and dissolved in culture medium immediately prior to use for studies in cells.

### Cell culture

T22-RGCΔFosLacZ cells, i.e. the murine prostate-derived cell line T22 stably transfected with a β-galactosidase gene reporter for p53 activation [51] (a kind gift from Xin Lu, Ludwig Institute for Cancer Research, Imperial College School of Medicine at St Mary's, London, UK), the human melanoma cell line ARN8 stably transfected with RGCΔFosLacZ [90] (a kind gift from J. Blaydes, University of Dundee, Dundee, UK) and its parental cell line A375 (no. CRL-1619, ATCC, Manassas, VA, USA) as well as human normal dermal fibroblasts (HNDFs; no. C-12300, PromoCell, Heidelberg, Germany) were cultured in Dulbecco's Modified Eagle's Medium (DMEM; no. SH30243.01, HyClone Laboratories, South Logan, UT, US) supplemented with 10% fetal bovine serum (FBS; no. SV30150.03, HyClone Laboratories) and 1% penicillin / streptomycin (P/S; no. SV30010, HyClone Laboratories). Human normal epithelial melanocytes (HNEMs; no. C12402, PromoCell) were grown in Melanocyte M2 Basal Medium (no. C-24300, PromoCell) without serum and antibiotics. The human colon carcinoma cell lines RKO BRAF^V600E/V600E/+^, RKO BRAF^−/−/+^ [55], RKO p53^+/+^, RKO p53^def/def^ [91], HCT116 p53^+/+^ and HCT p53^def/def^ [92], each kindly provided by Bert Vogelstein (Johns Hopkins University, Baltimore, MD, USA), as well as the human melanoma cell line HT-144 (no. HTB-63, ATCC) were grown in McCoy's 5A medium (no. M9309, Sigma-Aldrich) supplemented with 10% FBS, 1% P/S, and 1.5 – 3 mM L-glutamine (no. SH30034.01, HyClone Laboratories). RKO and HCT116 cells designated p53^def/def^ do not express isoforms of p53 which contain the first transactivation domain due to replacement of the first codon present in exon 2 with a resistance marker gene [91, 92]. It should be noted, however, that the modified gene present in these clones still encodes all isoforms containing a truncated N-terminus, e.g. Δ40p53 and Δ133p53 isoforms [93]. The human melanoma cell line SK-MEL-28, a kind gift from Stig Linder (Karolinska Institutet, Stockholm, Sweden), was cultured in Minimum Essential Medium Eagle (MEME; no. M5650, Sigma-Aldrich) supplemented with 10% FBS, 1% P/S and 2 mM L- glutamine. The human melanoma cell line Colo 829 (no. CRL-1974, ATCC) was grown in Roswell Park Memorial Institute medium (RPMI; no. SH30027.01, HyClone Laboratories) supplemented with 10% FBS and 1% P/S. No additional selenium source beyond FBS was used in the culturing of any of the cell lines, unless stated otherwise. All cell lines were confirmed free of mycoplasma, using the MycoAlert mycoplasma detection kit (no. LT07-418, Lonza, Rockland, ME, USA).

### Cell-based screen for wt p53 activators

A total of 20,000 compounds from ChemBridge (San Diego, CA, USA) were tested at a concentration of 10 μM in the ARN8 cell line stably expressing RGCΔFos-LacZ. After 18 hours of treatment, p53 activation was quantified by measurement of β-galactosidase activity as described [94]. Compounds were ranked on their ability to activate p53 as well as for drug-like chemical properties and other relevant information available on Scifinder and PubChem. p53 activation was confirmed in T22-RGCΔFosLacZ cells (data not shown). Other hit compounds from this screen will be reported elsewhere.

### p53-dependent transcription measurements

p53-dependent transcription was assessed by measurement of β-galactosidase activity in ARN8 cells using the p53-driven reporter plasmid RGCΔFos-LacZ as described previously [94]. Cells were treated at the indicated concentrations for 16 hours and β-galactosidase activity was normalized to protein concentrations.

### Western blotting

Samples were prepared as described previously [95] and analyzed with 4–12% and 12% precast SDS-PAGE gels (no. NP0321, NP0322 and NP0342, Invitrogen, Carlsbad, CA, USA) according to the manufacturer's instructions. Levels of scanned films (no. 28906837, GE Healthcare, Buckinghamshire, UK) were adjusted in Adobe Photoshop CS4 Extended in accordance with the guidelines for the proper handling of digital image data given in [96].

The following primary antibodies were used: anti-extracellular signal-regulated kinase 1/2 (ERK1/2) (no. 9102, Cell Signaling, Danvers, MA, USA; a kind gift from Leonard Girnita and Claire Worrall, Karolinska Institutet, Stockholm, Sweden), anti-glyceraldehyde 3-phosphate dehydrogenase (GAPDH; no. ab8245, Abcam, Cambridge, UK), anti-NAD(P)H:quinone oxidoreductase 1 (NQO1; no. sc-32793, Santa Cruz, Heidelberg, Germany), anti-Nrf2; no. ab62352, Abcam), anti-p21 (118) [97], anti-p53 (DO-7) [98], anti-phospho-ERK1/2 (no. 9101, Cell Signaling; a kind gift from Leonard Girnita and Claire Worrall) and anti-phospho-histone H2AX (Ser 139) (γ-H2AX; no. 05-636, Millipore, Molsheim Cedex, France). Horseradish peroxidase (HRP)-conjugated secondary antibodies were rabbit anti-mouse (no. P0161, Dako, Glostrup, Denmark) and swine anti-rabbit (no. P0211, Dako).

### Cell viability assay

Tests of cell viability were assessed using colorimetric SRB assays described by Skehan and colleagues [99]. Cells were seeded at low density in a 96-well plate, using the first column as blank control. After 72 hours of treatment, growth media were replaced with ice-cold PBS, and ice-cold TCA was added at a final concentration of 10% (w/v). After incubation at 4°C for 1 hour, plates were washed several times with tap water and air-dried. Subsequently, plates were stained with 0.4% SRB (w/v) in 1% acetic acid (v/v) for 30 min at room temperature (RT). Plates were de-stained by four sequential washes in 1% acetic acid. After air-drying, stained proteins were solubilized with 10 mM unbuffered Tris. Plates were shaken at medium speed for 5-30 min and absorbance was measured at 570 nm in a VersaMAX microplate reader (Molecular Devices) using the SoftMax Pro software.

To determine the effects of BSO, ARN8 cells were seeded at 200,000 cells per T25 flask in the presence of BSO [0.25 mM] or vehicle (sterile-filtered MilliQ water) for 72 hours. Afterwards, cells were trypsinized and each 10% of the cells transferred to a separate vial for determination of intracellular GSH levels (see separate subsection below). The remaining cells were counted and seeded at 500 cells per well into 96-well plates. After cell attachment, approx. 2 hours after seeding, MJ25 or auranofin were added at the indicated concentrations for another 72 hours. Cell viability was assessed by SRB assay as described above.

### Clonogenic assay

Cells were seeded at 10,000 cells per well in 6-well plates and treated on the following day as indicated. When compounds were combined, they were added simultaneously to the cells. After 72 hours, compounds were removed, the cells washed twice with growth medium, and fresh growth medium was added. Cells were let grow for 4 to 13 days. Afterwards, they were washed twice with PBS and fixed with methanol/acetone (1/1) at −20°C overnight up to 3 days. Subsequently, plates were air-dried and stained with Giemsa solution (7.5% (cancer cell lines) or 15% (HNDFs) in PBS, respectively) for 5 min at RT followed by two washes in lukewarm tap water and air-drying.

### *In vitro* DNA alkylation assay

MJ25's DNA alkylating capacity was assessed according to methods described in [100]. In brief, supercoiled pHOT1 DNA was mixed with the respective compound in 50 mM sodium phosphate buffer (pH 7.0) and incubated at 24°C for 6 or 24 hours, respectively. DMEDA was added at a final concentration of 100 mM and the mixture was subsequently incubated at 37°C for 1.5 hours. Afterwards, samples were loaded on a 0.5% agarose gel (w/v) containing 0.5% ethidium bromide (v/v). Pictures were taken with the GelDoc system (Bio-Rad). Chlorambucil served as positive control.

### Determination of inhibition of purified TrxR1 and glutathione reductase

Activities of purified TrxR1 were assessed by the direct NADPH-dependent DTNB reduction assay [101] and juglone reduction assay [39]. For this, recombinant selenocysteine-containing rat (*Rattus norvegicus*) TrxR1 (15 nM; ≈20 U/mg) produced as described [102], was incubated with 250 μM NADPH, 0.1 mg/ml BSA, and various concentrations of compounds. All experiments were performed in triplicate in 96-well plates and analyzed using a Spectramax microplate reader (Molecular Devices) using the SoftMax Pro software. Reference samples were treated with vehicle instead of active compound and were set as 100% activity, and no enzyme controls were used as blank references.

For purified TrxR1 inhibitory activity, 15 nM of NADPH-reduced TrxR1 was incubated with compounds for 15 minutes at RT. Following this pre-incubation step, 2.5 mM DNTB was added to each well and the linear increase in absorbance at 412 nm was followed for 90 seconds. For comparison to GR inhibitory activity, 2 nM of NADPH-reduced GR was incubated with compounds for 15 minutes at RT. Following this pre-incubation step, 10 mM of GSSG was added to each well and the linear decrease in absorbance at 340 nm was followed for 90 seconds.

Irreversible inhibition of TrxR1 was determined incubating 300 nM NADPH-reduced TrxR1 in the presence of compounds (2% DMSO) for 90 minutes. Aliquots of inhibited enzyme were used in a TrxR1-DTNB activity assay to determine native enzyme activity. The inhibited enzyme samples were subsequently desalted over a 30,000 molecular weight cutoff desalting column and activity was again determined using the DTNB assay.

Differential TrxR1 activities were determined after a 90 minute incubation, using juglone as a Sec-independent substrate and the DTNB assay as a Sec-dependent activity control. For the juglone assay, 10 μl of sample was added to Tris-EDTA buffer containing 250 μM NADPH and 100 μM juglone, and NADPH consumption was followed at an absorbance of 340 nm. 10 μl of the same master mix was additionally used to confirm complete inhibition of the selenocysteine active site using the DTNB assay. Concentrations were used to fully inhibit the Sec-dependent redox activity of TrxR1.

### Determination of cellular TrxR1 activities

The “endpoint” Trx1-dependent insulin disulfide reduction assay was used to measure activities of TrxR1 in cell lysates, as described elsewhere [101]. Briefly, 10 μg protein of crude cell lysate was incubated in the presence of 1.3 mM NADPH, 275 μM insulin, 10 μM recombinant human Trx1 (kindly provided by Arne Holmgren, Karolinska Institutet, Stockholm, Sweden) and 12.5 mM EDTA in 50 mM Tris buffer (pH 7.5) for 40 minutes at 37°C in a 96-well plate. Following this incubation, 7.2 mM guanidine-HCl containing 1 mM DTNB was added to each well to stop the reaction and detect thiolate groups with DTNB. Absorption was thereupon read at 412 nm on a 96-well Spectramax microplate reader (Molecular Devices) using the SoftMax Pro software. Background absorbance values using lysates incubated in parallel in the same reaction mixture without Trx1 were subtracted, and activities were given as percentages of cells treated with vehicle alone. DMSO treated cells were used at 100% activity controls, with samples lacking Trx1 in the reaction mixture serving as blank controls.

### ROS determinations using DCF

Cells were treated as indicated and harvested by trypsinization, including floating cells in the analyses. After centrifugation at 1300 x *g* for 5 minutes the cells were washed once with PBS and spun down as above. Pellets were resuspended in PBS containing 5 μM of the non-fluorescent substrate DCF-DA and incubated at 37°C for 30 minutes, protected from light. After centrifugation as above cell pellets were resuspended in 500 μl PBS, transferred to 5 ml polystyrene tubes, and fluorescence of the product DCF was analyzed by two-dimensional flow cytometry using a Becton Dickinson FACScan. Results were analyzed using the BD CellQuest Pro software (San Jose, CA, USA).

### Determination of intracellular glutathione levels

Intracellular total glutathione (GSH + GSSG) levels in the cells were determined as described previously [103]. Cell lysates derived from ARN8 cells treated with BSO or vehicle as described in subsection “Cell viability assay” were used.

### Statistical analysis

Statistical analyses were performed in Microsoft Excel 2010 using an unpaired one- or two-tailed Student's t-test, respectively, as indicated in Figure legends.

## SUPPLEMENTAl MATERIAL FIGURE


