# Supplementary material for: Redox effects and cytotoxic profiles of MJ25 and auranofin towards malignant melanoma cells
Source: Oncotarget. 2015 May 12;6(18):16488–506. doi: 10.18632/oncotarget.4108 (PMC4599284; doi:10.18632/oncotarget.4108)
Supplement: Supplementary file 1 [file oncotarget-06-16488-s001.pdf]

Redox effects and cytotoxic profiles of MJ25 and auranofin towards malignant melanoma cells

Supplemental Material

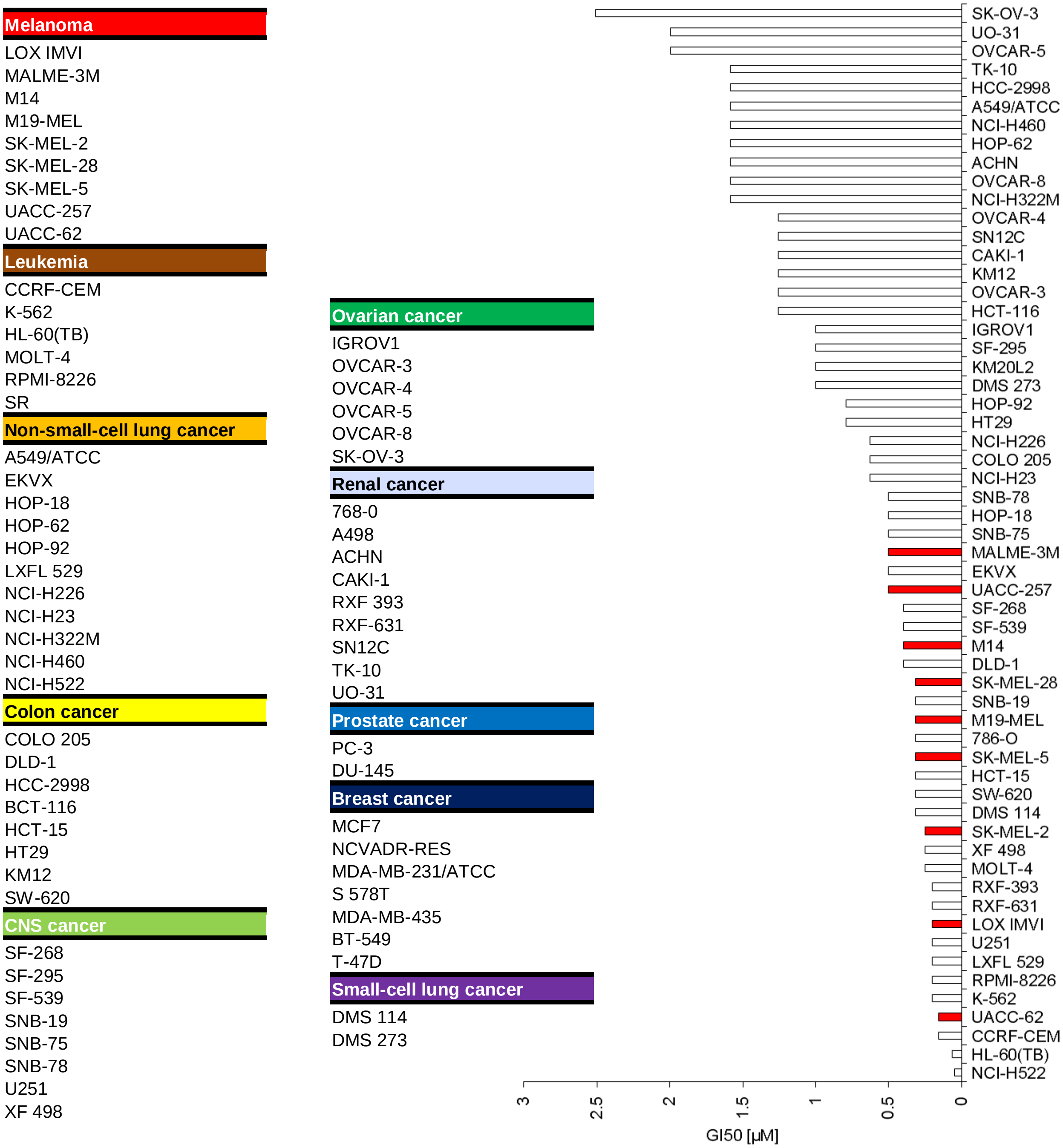

Supplemental Figure S1: Auranofin is very potent and efficiently kills tumor cell lines; melanoma cell lines are amongst the more sensitive ones (red bars). In connection with the Developmental Therapeutics Program (DTP) by the National Cancer Institute (NCI) at the National Institutes of

Health (NIH) auranofin's ability to inhibit cell growth and / or induce cell death after 48 hours of treatment had been determined by SRB assay. Data shown are derived from DTP's website (Cancer Chemotherapy National Service Center (NSC) number: 321521) (http://dtp.nci.nih.gov/index.html).
